# Typhoid Fever at Basingstoke

**Published:** 1905-10-28

**Authors:** 


### The Hospital.

A Journal of

The Medical Sciences and Hospital Administration.

Vol. XXXIX.—No. 996. SATURDAY, OCTOBER 28, 1905.

### TYPHOID FEVER AT BASINGSTOKE.

The deplorable ineptitude, or the still more deplorable negligence of the officials, or of the so-called "authorities," to whose tender mercies the care of the health of urban communities has been committed by the abounding wisdom of the Legislature, has received its latest illustration in the pleasant little town of Basingstoke, in Hampshire, where, out of a population of under ten thousand, 147 persons were stricken down with typhoid fever between September 18 and the 11th instant, and where seven of these died within the same limits of time. As displayed in a report of the meeting of the borough council, given in the *Hants and Berks Gazette* of the 14th instant, the history of the epidemic, although unfortunately only too intelligible, seems to exhibit a combination of folly, of ignorance, and of neglect of duty, for which, even in the history of provincial epidemics, it would not be easy to find a parallel. It appears that, towards the latter part of the month of July, the borough surveyor instituted an examination into the state of certain drains or sewers, and that these were plugged to keep back sewage from entering them while the examination was in progress. When it was concluded, the plugs were supposed to be removed, and the surveyor went for what seems to have been a month's holiday. He returned on the last day of August, and was at once informed by his subordinates of a mysterious overflow of sewage which had occurred during his absence, and was still continuing. Inspection discovered not only that one of the obstructing plugs had been left in position, but also that it was one which had been inserted without the surveyor's knowledge, and in a sewer which he had not thought it necessary to plug at all. It is said that the arrest of flow in one of those under inspection had not been complete, and that the additional plug in question had been fixed by a workman, under the authority only of a foreman, in one of the lateral feeders of the main channel. The sewage conveyed by this feeder, unable to follow its natural course, was compelled to find some outlet, and thus produced the overflow in question, which occurred at a point not far distant from one of the wells from which the town water supply was derived, and which seems to have found its way thither in the course of its meanderings. It does not seem to have occurred to anyone officially concerned that this communication between the town sewage and the town water supply might prove to be of considerable importance; and we gather from the report that the surveyor, having seen to the removal of the plug and of the obstruction produced by it, and by an accumulation of solid matters behind it, went home to bed sustained by a consciousness of rectitude and by a sense of accomplished duty. This was upon September 1, and on the 18th of the month two cases of typhoid fever were notified in the borough. On the 24th there were two more, and from this date the numbers increased to twelve, fifteen, and even seventeen in the course of a single day; until, as mentioned above, there were 147 cases in twenty-four days. During the present month the notifications have not exceeded four or five daily, so that there seems reason to hope that the epidemic has spent its force. The steed having been stolen, the local authorities appear to have left nothing undone in order to shut and fasten the stable door. The fever has not been confined to poor dwellings or to districts presumed to be insanitary, but has attacked people of good means and of prominent local position. Money seems to have been forthcoming in sufficient abundance, nursing has been organised, Dr. Farrar has been sent down in response to an appeal to the Local Government Board, and his report, when we obtain it two years or so hence, will doubtless prove to be an extremely interesting document. In the meanwhile, as is not unnatural, a sort of panic appears to have seized upon the inhabitants of the neighbourhood and to have rendered them unwilling to enter the town. Basingstoke is the market centre, so to speak, of a considerable agricultural district, and its trade is largely of the kind which such a position would imply. The desertion, even temporarily, of its shops and markets by the surrounding farmers and villagers is a matter of serious moment; and the Mayor and the chairman of the Health Committee of the Council have vied with one another in the enunciation of doctrines concerning the inocuousness of typhoid to the healthy, or to those who observe "proper sanitary precautions," which not every pathologist would be prepared entirely to endorse. Water and milk are to be boiled, and the country neighbours are informed that the fever is not "in the air," and that they may walk in the streets and do their shopping in complete security. We can only hope that they will listen to the voice of the charmer, and that the sorely afflicted town will not permanently suffer from their defection.

There are two important questions bearing upon the epidemic as to which we have so far received no information, but which will probably be explained in due time by Dr. Farrar. The first of these has reference to the source of the typhoid poison, the second to the absence of any warning from the local health office at the time when the cause and the direction of the overflow were discovered. The incubation period of typhoid has been variously stated by different observers, and so large a range as from five to thirty-five days has been assigned to it. At Basingstoke, as we have said above, the first cases were recognised on the eighteenth day after the presumable arrest of the contamination, and the great rush occurred about four days later. We have at present no information as to the class of dwellings which were drained by the obstructed sewer, or as to the health of the district from which it proceeded; but it would not be surprising to find that this district included common lodging-houses, to which "ambulatory" cases of typhoid are often supplied by tramps. The second question is a more serious one. Did the local medical officer of health know of the overflow, and of the direction in which it went? If he did not, how is his ignorance to be explained? and, if he did, how was it that no warning of a dangerous contamination of the water supply was given to the inhabitants?

